# Barriers and facilitators to the implementation of school-partnered collaborative type 1 diabetes interventions: a qualitative comparative analysis with school nurses to guide implementation

**DOI:** 10.48130/pedi-0026-0001

**Published:** 2026-06-22

**Authors:** Christine A. March, Elissa Naame, Sophia Alfinito, Jacquelin Rankine, Traci M. Kazmerski, Linda M. Siminerio, Ingrid Libman, Elizabeth Miller, Aaron R. Lyon

**Affiliations:** 1 Department of Pediatrics, University of Pittsburgh School of Medicine, 4401 Penn Ave Pittsburgh, PA 15224, USA; 2 University of Pittsburgh Dietrich School of Arts and Sciences, 139 University Place, Pittsburgh, PA 15260, USA; 3 Department of Medicine, University of Pittsburgh School of Medicine, 3550 Terrace St, Pittsburgh, PA 15261, USA; 4 Department of Psychiatry and Behavioral Sciences, 1959 NE Pacific St, Seattle, WA 98195, USA

**Keywords:** Type 1 diabetes, Implementation science, School nurse, Barriers, School intervention, Facilitators, Implementation, Qualitative, Pediatric, Care coordination

## Abstract

Though school-partnered interventions have the potential to improve health outcomes, data are limited to support their implementation for type 1 diabetes. The objective was to characterize barriers and facilitators (i.e., determinants) to implementing school-partnered collaborative care (SPACE), an intervention designed to improve care coordination, nurse education, and diabetes management in school. We conducted semistructured interviews with school nurses in two states. The interview guide was adapted from the Consolidated Framework for Implementation Research, which organizes determinants by the intervention, the individuals involved, the inner setting, (i.e., school district), the outer setting (i.e., external to the district), and the implementation processes. Major themes were summarized. We included 32 school nurses representing broad geographic regions, district sizes, and levels of nursing experience. School nurses reported that SPACE presented an advantage in care coordination compared with existing practice. Prominent barriers included (1) the intervention's complexity; (2) information sharing between schools and health systems; (3) high school nurses' caseloads, which may limit their availability; and (4) variable levels of parent engagement. Specific facilitators included (1) the intervention's adaptability and low cost, (2) high perceived need for interventions, (3) existing school resources that increase feasibility, (4) a focus on holistic outcomes which align with district priorities, and (5) relationships between school district nurses and leaders. Facilitators were perceived to be crucial to garnering buy-in from decision-makers within district leadership. The themes re-affirmed that school interventions for young people with type 1 diabetes are prioritized by school nurses. Implementation science frameworks can guide pre-implementation work and identify targets for implementation strategies which will mitigate barriers or leverage facilitators.

## Introduction

Despite modern advancements in treatments, children with type 1 diabetes and their families continue to face complex challenges. Fewer than one quarter of US youth reach glycemic targets, with nearly 30% living with a hemoglobin A1c > 9%^[1]^, placing them at increased risk for long-term complications^[2]^. Many families have reported barriers to accessing routine diabetes care, including associated travel and costs^[3]^. To supplement care from diabetes centers, one approach is to facilitate necessary health interventions through schools. Schools are a near-universal support for children, with approximately 85%–90% of enrolled US youth attending public school according to the National Center for Education Statistics^[4]^. For children with chronic conditions, school nurses are the cornerstone of their care in school^[5]^. They develop individualized health plans, administer medications, address emergencies, and educate other school staff about medical needs^[5]^. For these reasons, there are examples of school-based health initiatives helping to mitigate challenges for families in accessing the necessary services for asthma^[6]^ and mental health care^[7]^, though school-based diabetes interventions remain understudied and largely unproven^[8,9]^.

To address these challenges, we developed the School-PArtnered Collaborative carE (SPACE) program for children with type 1 diabetes^[10]^. Using human centered design (HCD) methods, we co-created the intervention by adapting a collaborative care model for Type 1 diabetes with multistakeholder partners. The overall goal of the program is to incorporate the school nurse into the multidisciplinary team. In virtual meetings of the nurse, parent(s), and diabetes care and education specialist, caregivers identify student-specific diabetes goals, develop plans for the school nurse to address management in school, and direct the family to any necessary referrals. The SPACE program is currently being piloted in 11 school districts^[11]^.

Although there is a strong rationale for school-based interventions for type 1 diabetes, interventions like SPACE are complex, as they involve coordination among families, schools and health systems. To design interventions which are both appealing to stakeholders and practical, HCD methods can be supported by adopting complementary methods from implementation science (IS)^[12]^. IS studies methods to bridge the research–practice gap and promote the uptake of evidenced-based practices/interventions^[13]^. The field relies on theories, models, and frameworks to plan for and evaluate the use of specific strategies to overcome implementation challenges and improve the success of an evidence-based practice in reaching a target population^[14]^. IS frameworks can help to systematically study barriers/facilitators prior to implementation and evaluate a newly designed intervention using HCD. In turn, methods from HCD may be used to select implementation strategies with the stakeholders^[12]^.

To date, there is limited literature applying IS to pediatric diabetes generally^[15]^ or specifically to diabetes in schools; most papers focus on routine aspects of care, rather than interventions, and lack comprehensive frameworks^[9,16]^. This creates a critical gap in understanding how diabetes interventions in school can be successfully implemented and translated to new settings with reproducible outcomes. Therefore, the overall goal of this study was to explore the barriers and facilitators which would affect the implementation of the SPACE program. A secondary goal was to construct a conceptual model to support clinicians and researchers seeking to partner with schools for diabetes interventions. We explored these concepts from the perspectives of school nurses, as they are the primary deliverers of diabetes care in school.

## Methods

We conducted a qualitative study using semi-structured interviews with school nurses as a component of pre-implementation planning for the SPACE program. SPACE will be implemented in a large, academic diabetes center (UPMC Children's Hospital of Pittsburgh). Our center serves approximately 2,500 children and young adults with diabetes across a wide geographic area in Pennsylvania (PA), West Virginia (WV), and Ohio. Details of the design and usability testing of SPACE^[10]^ and the protocol for the pilot feasibility trial are published elsewhere^[11]^. For this study, the research team comprised six physicians, one nurse, one psychologist, and one student. This team had expertise in type 1 diabetes clinical care, type 1 diabetes school care, school-based research, IS, and qualitative methods. A longitudinal community advisory board composed of nurses, diabetes providers, school nurses/administrators, and parents of children and adults with type 1 diabetes contributed to the study design. The University of Pittsburgh's Institutional Review Board deemed this study to be exempt (PRO22070051).

### Participants

We recruited school nurses from PA and a geographically proximal state, WV, which is partly served by our diabetes center. Eligible school nurses were included if they had at least one year of school nursing experience and worked in public school districts. We excluded school nurses who were retired or worked exclusively in private schools which do not receive federal funding and are thus not subject to the same legal requirements. Recruitment took place through the Pennsylvania Association of School Nurses and Practitioners (PASNAP), representing approximately 50% of school nurses (n *≅ * 1,000) across the state, and the email list server for the WV Department of Education Office of Student Well-being, which reaches all ~250 school nurses in the state.

To gain a deeper understanding of how implementation may vary in heterogenous settings, we (a) recruited no more than two nurses from any one school district; (b) recruited statewide, rather than being restricted to districts geographically proximal to our diabetes center; and (c) included nurses from two states with differences in their school district organization and school health policy. Pennsylvania contains 500 school districts organized by individual or clusters of municipalities overseen by the state Department of Health, whereas WV contains 55 county-based school districts overseen by the state Department of Education. In both states, school nurse caseload is set at one certified nurse per 1,500 students, though in WV, this only applies to kindergarten through to Grade 7. Over half (56%) of WV counties have a school nurse leader overseeing the health offices for the schools in that county. In PA, school nurses may report to another nurse, though they report more often to an educational administrator. School nurses are permitted to delegate all diabetes medications (insulin, glucagon) to other school staff who undergo training in PA; in WV, only glucagon may be delegated, and school nurses must be present for any child requiring insulin.

### Interview guide

We situated this study within the Consolidated Framework for Implementation Research (CFIR), a framework organizing barriers/facilitators (i.e., determinants) into five domains: The intervention, the processes for implementation, the individuals involved in the intervention, the inner setting (where the intervention is delivered), and the outer setting (everything external to where the intervention is delivered)^[17,18]^. As this was pre-implementation work contributing to the design and testing of a new intervention, the interview guide and analysis focused on four of the five domains (intervention, individuals, inner setting, and outer setting). Interview questions were adapted from the CFIR guidebook for diabetes interventions in school, piloted in the first three interviews, and modified to ensure clarity. The study's definitions within the framework and sample interview questions are included in Table 1; the full interview guide is available in the online supplement.

**Table 1:** CFIR domains applied to this study with sample interview questions.

CFIR domain	Definition for this study	Sample interview questions
Intervention	SPACE or similar collaborative intervention	How well do you think the collaborative care model (SPACE) would meet the needs of your students with type 1 diabetes?
Individuals	Participants involved in the intervention (e.g., school nurses, parents)	Who are the decision-makers in establishing changes or new programs for your school's approach to diabetes management?
Inner setting	School district where SPACE would be implemented	What factors help promote your school district's ability to embrace improvements in diabetes management?
Outer setting	Entities external to the district (e.g., neighboring school districts, local health systems, school nursing organizations, policies/regulations)	How do local, state, or national policies or regulations play a role in adopting new interventions?
Implementation processes	Not assessed	Not assessed

### Study procedures

School nurses provided verbal consent to complete the interview. Interviews were conducted by a trained qualitative researcher (CM) using the Zoom videoconferencing platform between January 2023 and December 2023. Interviews lasted 45–75 min and were conducted outside of school hours. Interviews were audiorecorded, transcribed, and de-identified. Prior to each interview, school nurses answered questions about their nursing and school workplace backgrounds. We distributed questionnaires via a personalized link to track responses.

### Data analysis

We summarized the participants' characteristics with descriptive statistics and compared them by state using Fisher's exact test. Interviews and qualitative analysis occurred concurrently so that the interviews could be stopped when we achieved content saturation. We used a thematic analysis^[19]^ with a directed, deductive approach using the available CFIR resources, including a codebook composed of CFIR constructs by domain^[20]^. Code definitions were minimally adjusted to the study context (e.g., type 1 diabetes, schools). Two trained reviewers (EN, SA), under close supervision by a trained qualitative researcher (CM), independently reviewed the first five transcripts, applied the codebook, compared the coding, and refined code descriptors for further analysis. The reviewers applied the final codebook to each transcript and met regularly to review the coding, facilitate consensus, and identify emerging themes^[21]^. Themes were derived from all transcripts, though separate excerpts were reviewed by state for each theme to evaluate for possible differences in school nurses' perspectives. Emerging themes were reviewed with the community advisory board to assess how they resonated with experiences and expectations. The final themes were aligned with the CFIR-defined domains and largely condensed between the two states, as there were minor differences observed in the responses from school nurses in PA and WV, except as noted in the results. Representative quotations are included with the individual context provided, including the state, years worked as a school nurse, and geographic region. As a final step, the research team integrated final themes into the CFIR determinant framework to develop a conceptual model outlining how the progression of CFIR domains influence adoption of the innovation, overlaying relevant CFIR constructs within each domain from our results. Dedoose version 9.2.005 was used for the coding process and data management.

## Results

We interviewed 32 school nurses in PA (*n *= 20) and WV (*n *= 12) to achieve thematic saturation. Characteristics of the school nurses and districts are included in Table 2. Nearly all participants identified as female (97%), and all identified as non-Hispanic white. All participants held a minimum of a bachelor's degree. The participants covered one to three schools, with the highest proportion reporting a caseload of 1,001–1,500 students. Nurses represented predominantly suburban and rural districts, similar to the state demographics, with a range in the district size (~680 to 24,000 students), indicating the inclusion of participants representing small, medium, and large districts. Less than half of the school nurses (38%) had a school nurse supervisor, which was more commonly reported among the WV nurses (58%) compared with those in PA (25%). Themes and representative quotations are included in Table 3.

**Table 2:** Characteristics of interview participants and represented school districts.

Characteristic	PA (*n* = 20), *n* (%)	WV (*n* = 12), *n* (%)
Highest degree		
Bachelor's degree	6 (30)	8 (67)
Master's or Doctorate	14 (70)	4 (33)
Years as a school nurse		
< 10 years	7 (35)	7 (58)
≥ 10 years	13 (65)	5 (42)
Geographic setting		
Rural/town	5 (25)	9 (75)
Suburban	13 (65)	2 (17)
City/urban	2 (10)	1 (8)
Number of schools covered		
1	7 (35)	6 (50)
2	7 (35)	4 (33)
> 2	6 (30)	2 (17)
Grades covered^a^		
Elementary (K–5)	13 (62)	8 (38)
Middle (6–8)	10 (62)	6 (38)
High (9–12)	13 (81)	3 (19)
Student caseload		
< 750	5 (25)	6 (50)
750–1,000	6 (30)	3 (25)
1,001–1,500	9 (45)	3 (25)
Student caseload (students with diabetes in the past 5 years)		
< 5	5 (25)	3 (25)
5–10	8 (40)	7 (58)
11–15	4 (20)	0 (0)
> 15	3 (15)	2 (17)
^a^ Nurses could select more than one grade distribution.		

**Table 3:** Themes organized by CFIR domain with representative quotations.

CFIR Domain	Theme (CFIR constructs/codes)	Representative quotation(s)
Intervention	Theme 1: SPACE may be timesaving by proactively addressing concerns (innovation's relative advantage; innovation's cost)	"*We have these students in school for 12 years. If we can establish a really good program and a really good relationship, and really good expectations on what's going to happen with their care in school, a consistent message stays all the way through, then it alleviates a ton of stress, a ton of worry, a ton of concern, all from both the educators, the staff, the teachers, from our parents at home, and, ultimately, us.*" (PA Nurse 18, < 5 years, suburban region)
		"*I think we need more communication between the nurses and the providers and parents. We all need to be on the same page. I like updates. If a student goes to a doctor for a visit, I would like to have an update on what they discussed. How are they doing? It's like, you don't hear any of that feedback. I feel, as a nurse trying to take care of these students, I need to know that feedback.*" (WV Nurse 8, < 5 years, rural region)
Intervention	Theme 2: Remaining hurdles: The intervention's complexity and evidence base (innovation's evidence base; innovation's complexity; innovation's adaptability)	"*Well, the cost of it, obviously, how much time it's going to take, how it's going to benefit my students, and how much time is it going to take up to implement and then keep running.*" (PA Nurse 8, 5–10 years, suburban region)
	Subtheme: Students who may benefit	"*If we introduce that care model, I really feel [it] would benefit the ones where we're struggling… the kids that are coming in the 300, 400s. I would say it would probably be more of our at-risk students that we would focus because we're already spending—the nurses are already spending a lot of time with these students.*" (WV Nurse 1, 5–10 years, rural region)
	Subtheme: Promoting adaptability	"*We just have to structure it around our time, when we're not busy with medications or diabetes care, that sort of thing, other treatments. It would be based on the building and the student, as to what the rest of the schedule looks like for the day*." (PA Nurse 5, 11–20 years, suburban region)
Outer setting	Theme 3: Relationships between school districts can be influential (external pressure; partnerships and connections; critical incidents)	"*I definitely think that if another school district's doing something and it's working for them, and we see that—I think that that would be for sure something that our district would be on board with, with giving a try.*" (PA Nurse 1, 11–20 years, suburban region)
	Subtheme: These relationships may be less prominent in larger school districts.	"*I would say not so much what other counties or school districts near us. Probably we would want to know more like how other larger places [city] have done it because sometimes it's a little easier to look that way. We have a little bit of different structure in our county. Just because we have multiple people and we do have a supervisor. Some counties only have one or two nurses. They don't have a nursing supervisor. It's very different for them.*" (WV Nurse 11, 5–10 years, rural region)
Outer setting	Theme 4: Concerns about privacy limit interactions with the health care system. (policies and laws; partnerships and connections)	"*I think we could do a better job of outreach to their diabetes educators, though there's no smooth way to do that.*" (PA Nurse 16, >20 years, suburban region)
	Subtheme: Communication may be smoother with consistent points of contact.	"*We just call [the clinic nurse]. Pretty much she just tells us what we need to do to make sure we're all on the same page. She could even pull up the information from the student side. She works really well.*" (WV Nurse 10, 11–20 years, rural region)
Inner setting	Theme 5: Interventions must align with leadership priorities (mission alignment; compatibility; relational connections)	"*I just think if it was just presented in the right fashion and [I] explain the details of…how this was going to look and how it would benefit the students, then I think they would be very supportive and be there to help in any way necessary.*" (PA Nurse 9, >20 years, suburban region)
		"*Usually, our coordinator of health services, whatever she wants, she gets. They place safety first for all of our kids at the level of superintendent and down. Usually they know that healthy children are the best kind of learners because they're feeling good enough to come to school, to pay attention in class, and that maintaining that level of health is important to their education.*" (WV Nurse 12, 11–20 years, urban)
		"*It would be—this is in line with what IDEA [Individuals with Disabilities in Education Act] is trying to demand of us, that we are a team, and we're going to help this kid be safe and educated. At that point, you're just like, 'Listen, this is a special needs kid.' We actually have to just do the thing that it takes to do it right. End of story.*" (PA Nurse 12, 5–10 years, urban region)
Inner setting	Theme 6: The school district culture is generally supportive of diabetes interventions (culture; incentive systems)	"*Their diabetes isn't going away. How can we help them get through school? If there's any changes or any studies or anything like that. That can improve the health and education of our students. They're very willing to say, 'Okay, show me what ya got. What's out there? What's the new and greatest thing to help these students?'"*. (WV Nurse 1, 5–10 years, rural region)
	Subtheme: School staff may need more education about diabetes care.	"*I think when a student shows up on a teacher's caseload or another—cafeteria or recess, a non-nursing staff member's caseload that it is like you're starting from the very bottom of teaching them, unless, for some reason, they've had a recent interaction with a student with diabetes. At large, staff is not prepared at all, but nursing, in my opinion, has been.*" (PA Nurse 7, 5–10 years, suburban region)
Inner setting	Theme 7: Existing programs/infrastructure enhance feasibility (compatibility; structural characteristics; resources)	"*With Zoom anymore, we can build that in and have the student here and Zoom with the parents. I think it's pretty workable. It's almost like any more you can make anything work*." (PA Nurse 17, > 20 years, rural region)
		"*It's called Student Assistance Team and it's usually for kids who maybe don't have an IEP or a 504, but they're having some kind of issue, whether it's with attendance, whether it's with behavior, whether it's with a health problem that hasn't gotten to the point where it needs a 504 or maybe we're thinking that it could need a 504. We go that route and visit first, and try to establish something and then if we feel like it needs to go beyond that, then we go beyond it.*" (WV Nurse 3, > 20 years, rural region)
Individuals	Theme 8: School nurses are highly motivated yet face competing demands (capability; opportunity; motivation; implementation leads)	"*Even just the chronic health care, I really love the work that we do here with our kids with diabetes because I feel like we make a big difference in their lives and the lives of their families, and I love that we get to see them all the time and contribute to how their life will be in the future. That's why I wanted to be here. That's why I wanted to do what I do.*" (PA Nurse 2, 5–10 years, rural region)
		"*There's often a lot—there are other things going on in schools besides diabetes management. That might be a struggle in some situations.*" (WV Nurse 6, 11–20 years, rural region)
Individuals	Theme 9: Parent engagement is variable within districts (implementation recipients; opinion leaders)	"*Some parents, they seem to be involved, but yet they're distant, I guess is—they come the first day of school, and then there's never any other good rapport, communication, even if email or try to call back and forth. I've watched this change over the years*." (PA Nurse 17, > 20 years, rural region)
		"*The parents that I have worked with, they want to be involved with the school health nurse. They want to make sure that the school health nurse is knowledgeable of all the orders. They want us to have access. We've never had a problem of saying, 'Do you mind if we have to call? We would, of course, let you know, but if we need to call and ask a question or fi—or need some record or something of a visit.' We've never had an issue. The parents are glad that we are paying attention to their children because sometimes a [student with diabetes] can walk around, and you wouldn't even know that anything was going on.*" (WV Nurse 2, <5 years, rural region)

### Intervention

#### Theme 1: SPACE may be timesaving by proactively addressing concerns

The primary advantage was the potential for greater teamwork on behalf of the students. Some described a huge disconnect between doctors and schools, which often leaves school nurses unclear on students' needs if the parent does not communicate well. School nurses appreciated the potential for collaborative interventions to offer more support to students with multiple needs (e.g., diabetes, mental health, absenteeism), create a system for parent accountability, and improve inefficiencies in care. One nurse stated, "*I feel like it would just behoove everybody if we could all just be able to communicate… If all of us were on the same page, there'd be less of finger pointing and more just, like, let's help this child*." (WV Nurse 12, 11–20 years, urban region). Other benefits were perceived to be the low cost of the intervention and its virtual format.

#### Theme 2: Remaining hurdles: The intervention's complexity and the evidence base

School nurses' concerns included the existing evidence to support SPACE, identifying clear needs for SPACE to support the time commitment, and the complexity of coordinating an intervention involving the school, the clinic, and home. To overcome these, school nurses shared different recommendations. For example, they suggested criteria for which students would benefit most from SPACE (e.g., significant glycemic variability, co-existing learning/mental health needs). They identified important outcomes to judge the effectiveness of the intervention, including students' glycemia, wellbeing, and inclusion in school activities. Lastly, they discussed ways to promote the adaptability of the intervention, such as flexible scheduling before or after school.

### Outer setting

#### Theme 3: Relationships between school districts can be influential

School nurses identified the importance of relationships between schools and other entities, such as neighboring school districts or school nurse communities. Communication between these entities can help support the implementation in different ways. For example, some participants reported regular meetings between district leaders to, "*see who's doing what, to get ideas, and to kind of piggyback off each other*" (PA Nurse 14, 5–10 years, suburban region). The COVID-19 pandemic shone a light on that, as districts tried to align recommendations to help justify decisions for parents. Others believe that adopting a new diabetes intervention would be appealing to promote their district. One nurse stated, "*The benefit is that we would be able to be one of the first to implement something like that, and that's always a value. Bragging rights, if you will, for the superintendent*." (PA Nurse 20, > 20 years, rural region).

This cross-district influence also existed among nurses who identified text chains, email listservs, or standing meetings, for example, through county-level school nurse associations in PA. These gatherings provided an opportunity to talk about what is and is not working in schools and get new ideas. These were identified as potential forums to distribute information about SPACE and generate interest. Notably, these external relationships were less prominently reported by school nurses in WV, where the county-based district system leads to fewer districts covering a wider geographic area. One nurse illustrated this by sharing, "*The school system, I've never seen anything like it in my whole entire life. [This] county will do something that [another] county won't do... They don't care. It's just whatever that county in that board wants to do*." (WV Nurse 8, <5 years, rural region).

#### Theme 4: Concerns about privacy limit interactions with the health care system

Relationships with treating diabetes clinicians were perceived differently, summarized by one nurse who shared, "*It has seemed to be difficult to have direct communication with the medical decision-makers*." (WV Nurse 6, 11–20 years, rural region). They perceived that clinicians may limit information-sharing out of concerns about privacy and confidentiality, despite the provision of medical orders and the school's internal process for parents' signed release. Communication may be smoother if there is a consistent point of contact. Nurses indicated that this relationship should be addressed and bolstered in the SPACE intervention.

### Inner setting

#### Theme 5: Interventions must align with leadership priorities

Specific to the school setting, nurses discussed the ways in which school leaders impact the district's support and existing resources for SPACE. Though often aligned, school nurses distinguished their administrators' support for health staff and their support for interventions. For example, one nurse commented that leadership was very supportive of her position, as the school nurse is the only medical person in a sea of educators, which meant that the administrators often deferred to her expertise in health decisions and would embrace change. Others discussed a positive rapport with their leaders, yet described resistance to change, which had led to prior school nurse-driven interventions being rejected. This was illustrated by one PA nurse who shared, "*I do feel like our administration is pretty set in their way of their values. We have some that are open to listening and then others that are very closed-minded, so it just depends. At the same token, [Superintendent] does care about the students and he does support them, so I don't want to give the impression that he wouldn't care. It would be a change. I think it would take a little bit more umph to justify to him why we would make a big change*" (PA Nurse 18, < 5 years, suburban region). School nurses recognized leaders as more supportive if they shared a student-centered, holistic view, where equal emphasis was placed on education and health to support optimal learning. As such, nurses suggested appealing to leaders with an understanding of how the SPACE intervention may impact both health and educational goals.

#### Theme 6: School district culture is generally supportive of diabetes interventions

Downstream of the leaders, nurses appreciated that the leaders set the tone for the district's culture in how students with diabetes are supported by teachers and other staff. Many school nurses indicated wide support among staff, stating, "*I think people realize the severity of a diabetic crisis and they don't want anybody to experience that*." (PA Nurse 1, 11–20 years, suburban region). Even though the teachers are all willing to come forward to ask questions, they are generally viewed as being unprepared to assist students and in need of additional education. Nurses expressed a desire for SPACE to create more uniformity for lay staff who may be involved in diabetes care.

#### Theme 7: Existing programs/infrastructure enhance feasibility

Another factor which may influence the school's adoption of SPACE relates to the available infrastructure. School nurses frequently noted that SPACE was similar in some ways to existing school programs. For example, school nurses drew parallels between SPACE and a student assistant program in PA, which puts together a multidisciplinary team of administrators, nursing, guidance, teachers, and outside agencies to assist students who need special support. This program was not required in WV. However, others discussed students receiving mental health or developmental services in school, which established a precedent and helped schools understand how they could feasibly incorporate SPACE into the school day.

Physical infrastructure, such as more widely available tablets, computers, and video-conferencing platforms, was also seen as a facilitator of the SPACE intervention. One nurse shared, "*Depending on how many people meet, we have conference rooms available that we could meet together on our end. All of us—all of us have laptops. Even the students have laptops. They might be jumping on with us or so. We have space and we have technology. I don't see that as a barrier*." (PA Nurse 6, > 20 years, suburban region). This was attributed largely to changes in school resulting from the COVID-19 pandemic. Another nurse shared, "*I think since COVID, our IT department is so ready for any kind of meeting like this that we have that it's not an issue either. We all have capabilities with our computers*." (PA Nurse 3, 11–20 years, rural region).

### Individuals

#### Theme 8: School nurses are highly motivated yet face competing demands

Generally, school nurses described strong motivations to support students with type 1 diabetes which, in turn, benefits their nursing practice. School nurses recognized that children spend a great proportion of their day in school, which is a lot of time to spend managing diabetes. This is a challenge for students who have chronic hyperglycemia. One nurse stated, "*Those three [students] can take your whole day. One may be so poorly controlled at home that you're trying to play catch up in the school day, and knowing that when you send them home, we're going to start that cycle all over again*." (WV Nurse 1, 5–10 years, rural region). Even if diabetes is well-managed, younger students need constant supervision, which adds to the school nurses' workload. Nurses reported that SPACE may help them feel more included in a diabetes team which recognizes their training and teaches additional approaches to help students manage better in school and at home. One nurse stated, "*I want my kids to have long, healthy lives, and whatever I can do to serve them, I'm going to do that*." (PA Nurse 4, <5 years, suburban region). At the same time, momentum to institute SPACE may lag because of competing demands, particularly if there are few students with diabetes in any given district and/or the nurses have high student caseloads.

#### Theme 9: Parent engagement is variable within districts

School nurses shared variable insights about parents, who would also be key participants in SPACE, displaying both empathy and frustration. Parents were described by some as stressed, overwhelmed, distant, and the biggest obstacle. Parents may be present at the beginning of the school year, only to become difficult to get in touch with. Some nurses also expressed concerns regarding parents or secondary caregivers' comprehension of diabetes or familiarity with technology, which could interfere with their ability to join a virtual meeting. Those who had strong bonds with parents suggested that parents would welcome SPACE. One nurse stated, "*I think that they would respond well because that way, they have a team of people that are caring for their child, and everybody is in communication with everyone. So, everyone's in the know*." (WV Nurse 5, < 5 years, rural region). They felt that parents might appreciate better communication, reduced school absences, and sharing responsibility with another caring adult.

## Discussion

In this qualitative study, school nurses shared their perceptions of the broad array of factors which would influence the implementation of SPACE. Though studies have reviewed barriers and facilitators to providing diabetes care in school generally^[16,22]^, this study is among the first to describe the multilevel determinants which should be considered when implementing a school-based intervention for children with diabetes. We organized our findings within a commonly used determinant framework in the IS literature, CFIR, allowing us to characterize and define factors related to the intervention, the individuals involved, the school (inner) setting, and external to the school (outer) setting. Though our study was focused on a specific intervention, the information learned may be broadly applied to other school-partnered interventions for children with diabetes with the use of the CFIR framework.

Several of our themes aligned with prior reports of CFIR constructs affecting the implementation of participatory interventions in school, though those were for health prevention and other chronic conditions (e.g., asthma). Specifically, the importance of leadership engagement^[23−27]^, alignment with institutional priorities^[24,27]^, understanding the perceived need for the intervention^[25,26,28]^, existing infrastructure/resources to promote readiness^[28]^, and parent engagement^[23,26]^ have all been identified as barriers or facilitators in past intervention studies. These commonalities across different school-based health interventions support the validity of our findings and provide avenues to strengthen future interventions' implementation by informing the design of implementation strategies. For example, specific IS initiatives can be used to foster leadership engagement to promote a specific evidence-based practice within the school environment, though this has been evaluated to a limited extent in schools to date^[29,30]^. Applying such IS concepts may be beneficial when planning for school-based diabetes interventions.

Our findings also reaffirmed the potential value for diabetes interventions in school. Despite the barriers, we found that school nurses placed high priority on interventions to support students with type 1 diabetes. Possible drivers include the rising incidence of type 1 diabetes^[31]^, the intensive nature of therapy, the high social needs of many children^[32]^, and frustrations over the time spent coordinating care with parents and health systems^[33]^. These pressure points can inform a data-driven approach when engaging with school nurses and leadership as to how the intervention may address these complex challenges and improve school care. To garner buy-in, interventions should be multicomponent to address several concerns with measurable impact, such as with student management, care coordination, communication, and education/training for school staff^[16]^.

Even with active school participation, parent engagement remains a concern. Prior studies have generally found a high degree of parent-reported acceptability for type 1 diabetes interventions in school^[34,35]^, although these included small cohorts (23–30 families) and predated many modern advancements in diabetes management. In a more recent study, we explored the drivers of parent engagement in school health decisions for their children with type 1 diabetes, finding that demanding parent schedules did not uniformly negatively impact their engagement^[36]^. Rather, many parents saw the nurse as an active partner, which would strengthen the need for collaborative interventions. Fostering positive relationships, emphasizing consistent and frequent communication, offering services (e.g., transportation, child care), and actively incorporating parents' feedback and priorities are potential solutions to address parents' barriers to participation^[37,38]^.

A unique aspect of our study is the integration of data from two different states, PA and WV, which have slightly different policies affecting school district organization, school health staffing, and delegation of diabetes care in school. There is growing interest in studying policy within IS research^[39]^. In the case of type 1 diabetes, state policies for school health provide an important context which may affect the implementation of interventions. Indeed, as many school-based diabetes research studies focus on a narrow geographic region, generalizability has been a common limitation, given the known state-to-state variability. Our findings provide a case example for how policies may or may not alter school nurses' perspectives on type 1 diabetes interventions. For example, the themes were similar when considering their motivations and the need for interventions to support this population. However, they described differences in their leadership structure (e.g., it includes a nurse or not) and the presence of state-mandated student assistance programs. A novel finding was that communication between neighboring school districts may differ regionally and can influence decisions. This CFIR construct of "external pressure" is an uncommonly reported outer setting variable, with one study finding that relationships between schools may help facilitate an intervention's uptake by promoting trust and strengthening planning^[27]^. Altogether, these differences suggest some degree of state-level variability, which can impact implementation processes. For example, in these two states, a plan to work with county-based school nursing organizations in PA to disseminate information about the SPACE intervention may be less effective in WV, where such organizations may be less active.

Using the implementation determinants identified in our themes, we developed a conceptual model to organize CFIR constructs within the four domains likely to influence the adoption of Type 1 diabetes interventions (Fig. 1). Consistent with other reports in the literature, our model demonstrates the crucial role of multilevel leadership engagement as the primary driver of both the school culture (i.e., climate) and the actions (i.e., citizenship behavior) of individuals involved to support the intervention's adoption^[40]^. In the outer setting, we depict the role of state policies (e.g., staffing requirements, medication delegation laws) and external relationships influencing district capacity and leadership engagement. Individual and inner setting factors further contribute to the implementation climate and leaders. Finally, adoption is also influenced by the intervention's characteristics. This model can serve as a "roadmap" for those seeking to implement school-partnered programs by providing a broad overview of important considerations to assess before or after the implementation.

**Figure 1:**
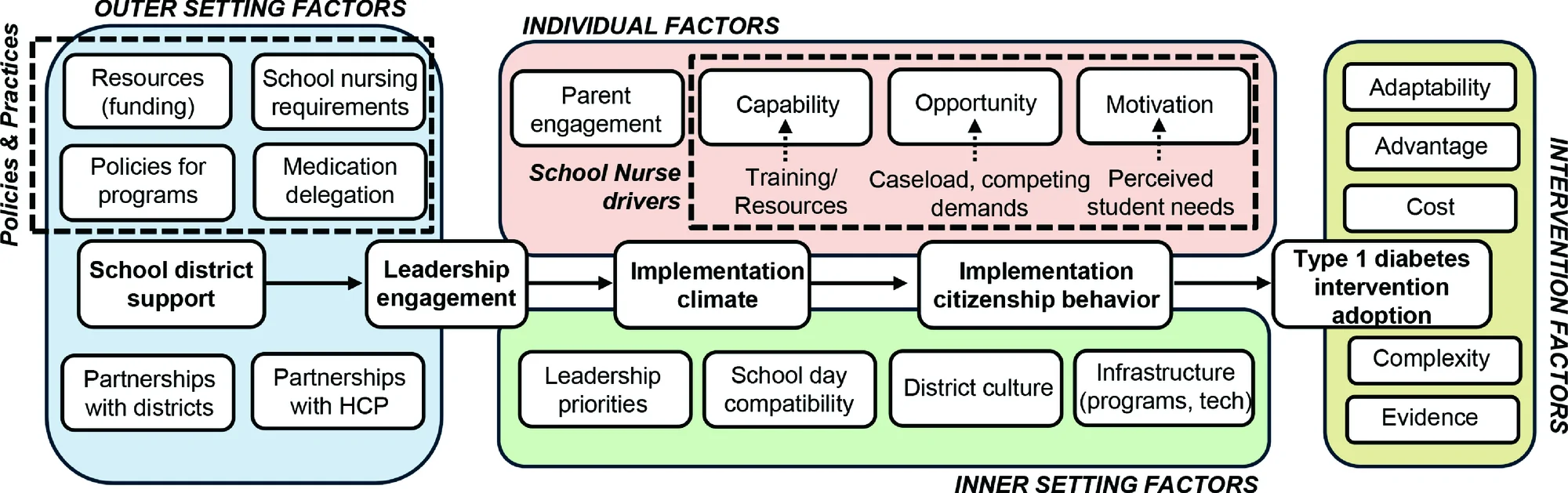
Conceptual model of CFIR determinants of the implementation, which may influence the adoption of Type 1 diabetes interventions in schools. Determinants are categorized by CFIR domains: Outer setting (external to the school district), inner setting (school district), individuals involved, and the intervention's characteristics. These determinants are hypothesized to influence district support, leadership engagement, the climate for implementation, identification of implementation leads, and ultimately adoption. This conceptual model can guide pre- and post-implementation assessments for school-based diabetes interventions. Abbreviations: HCP, healthcare providers.

The strengths of our study include the application of a determinant framework to study pre-implementation factors for diabetes interventions in school^[8]^. Additionally, our case study allowed for an exploration of how state-level policy may influence organizational readiness to adopt interventions. The limitations of our study include the focus on school nurses over other school staff, and individual rather than district perspectives. This was intentional, given the integral role of school nurses in diabetes care and the possibility for school nurses in different schools within one district to have highly variable experiences. None of our participants worked in the same schools. The proportion of rural and suburban districts in PA and WV was significantly different. This is a limitation as proximity to healthcare is likely to influence perspectives beyond just the policy context. However, our locale distribution reasonably represented each state. Less than 10% of the school districts in either state are classified as urban, and approximately 81% and 47% of school districts are rural in WV and PA, respectively. We did not include data from healthcare providers or parents on their ability to engage with schools, as these have been explored in separate studies. Lastly, as state policies for staffing and insulin delegation vary by state, future research should evaluate the hypotheses generated from our findings in a broader context.

## Conclusions

School-based interventions to assist families with children with chronic diseases are promising yet complex to implement. State-level policies may influence an intervention's adoption and reach. Using determinant frameworks to proactively study the barriers and facilitators can assist investigators with essential pre-implementation work to deliver complex school-partnered interventions when translating to new geographic settings.

## Data Availability

The datasets generated and/or analyzed during the current study are not publicly available because of ongoing secondary analyses but are available from the corresponding author on reasonable request. Interview guides and code books based on CFIR are publicly available online: https://cfirguide.org.

## References

[1] (2023). Longitudinal trends in glycemic outcomes and technology use for over 48, 000 people with type 1 diabetes (2016−2022) from the T1D exchange quality improvement collaborative. Diabetes Technology & Therapeutics.

[2] (2014). The diabetes control and complications trial/epidemiology of diabetes interventions and complications study at 30 years: overview. Diabetes Care.

[3] (2014). Prevalence of and disparities in barriers to care experienced by youth with type 1 diabetes. The Journal of Pediatrics.

[b4] 4National Center for Education Statistics. 2022. Digest of Education Statistics. U. S. Department of Education, Institute of Education Sciences. doi: 10.1037/e492162006-024 (Accessed on March 11, 2026)

[5] (2016). Role of the school nurse in providing school health services. Pediatrics.

[6] (2019). Effectiveness of school-based self-management interventions for asthma among children and adolescents: findings from a Cochrane systematic review and meta-analysis. Thorax.

[7] (2021). Rates of mental health service utilization by children and adolescents in schools and other common service settings: a systematic review and meta-analysis. Administration and Policy in Mental Health and Mental Health Services Research.

[8] (2015). School-based diabetes interventions and their outcomes: a systematic literature review. Journal of Public Health Research.

[9] (2021). Role of theories in school-based diabetes care interventions: a critical review. World Journal of Clinical Cases.

[10] (2025). School-partnered collaborative care (SPACE) for pediatric type 1 diabetes: development and usability study of a virtual intervention with multisystem community partners. JMIR Diabetes.

[11] (2026). School-partnered collaborative care intervention for young children with type 1 diabetes: a pilot and feasibility protocol. Contemporary Clinical Trials.

[12] (2021). Complementary approaches to problem solving in healthcare and public health: implementation science and human-centered design. Translational Behavioral Medicine.

[13] (2015). An introduction to implementation science for the non-specialist. BMC Psychology.

[14] (2015). A refined compilation of implementation strategies: results from the Expert Recommendations for Implementing Change (ERIC) project. Implementation Science.

[15] (2024). Implementation science and pediatric diabetes: a scoping review of the state of the literature and recommendations for future research. Current Diabetes Reports.

[16] (2022). Implementation of school diabetes care in the United States: a scoping review. The Journal of School Nursing.

[17] (2009). Fostering implementation of health services research findings into practice: a consolidated framework for advancing implementation science. Implementation Science.

[18] (2022). The updated Consolidated Framework for Implementation Research based on user feedback. Implementation Science.

[19] (2006). Using thematic analysis in psychology. Qualitative Research in Psychology.

[b20] 20CFIR Leadership Team. 2022. Consolidated Framework for Implementation Research: Qualitative Data. https://cfirguide.org/cfir-tools-templates (Accessed on January 19, 2026)

[21] (2005). Consensual qualitative research: an update. Journal of Counseling Psychology.

[22] (2022). Determinants of school diabetes care services and quality through the lens of the school nurse: a qualitative analysis. Advances in Nursing Science.

[23] (2010). Evidence-based mental health programs in schools: barriers and facilitators of successful implementation. School Mental Health.

[24] (2018). Barriers and facilitators to the implementation of physical activity policies in schools: a systematic review. Preventive Medicine.

[25] (2020). Factors affecting the implementation of a whole school mindfulness program: a qualitative study using the consolidated framework for implementation research. BMC Health Services Research.

[26] (2021). Facilitators and barriers to implementation of school-based telehealth asthma care: program champion perspectives. Academic Pediatrics.

[27] (2021). Evaluation of a school-based participatory intervention to improve school environments using the Consolidated Framework for Implementation Research. BMC Public Health.

[28] (2024). Relationship between the inner setting of CFIR and the delivery of the Healthy School Recognized Campus initiative: a mixed-methods analysis. Implementation Science Communications.

[29] (2025). Not getting better but not getting worse: a cluster randomized controlled pilot trial of a leadership implementation strategy. Implementation Research and Practice.

[30] (2025). Study protocol for testing the efficacy of the Helping Educational Leaders Mobilize Evidence (HELM) implementation strategy in elementary schools: a hybrid type 3 effectiveness-implementation randomized controlled trial. Implementation Science.

[31] (2020). Trends in incidence of type 1 and type 2 diabetes among youths—selected counties and Indian reservations, United States, 2002–2015. MMWR Morbidity and Mortality Weekly Report.

[32] (2024). Social determinants of health screening in type 1 diabetes management. Endocrinology and Metabolism Clinics of North America.

[33] (2020). Modern diabetes devices in the school setting: Perspectives from school nurses. Pediatric Diabetes.

[34] (2005). Improving students' diabetes management through school-based diabetes care. Journal of Pediatric Health Care.

[35] (2009). School-centered telemedicine for children with type 1 diabetes mellitus. The Journal of Pediatrics.

[36] (2025). Parent engagement in school health decisions for type 1 diabetes: barriers to and facilitators of collaboration and implications for intervention design. Diabetes Spectrum.

[37] (2013). Engaging parents in evidence-based treatments in schools: community perspectives from implementing CBITS. School Mental Health.

[38] (2014). Mental health interventions in schools in high-income countries. The Lancet Psychiatry.

[39] (2023). Four very basic ways to think about policy in implementation science. Implementation Science Communications.

[40] (2014). Aligning leadership across systems and organizations to develop a strategic climate for evidence-based practice implementation. Annual Review of Public Health.

